# Selenium Concentration Is Positively Associated with Triglyceride-Glucose Index and Triglyceride Glucose-Body Mass Index in Adults: Data from NHANES 2011–2018

**DOI:** 10.1007/s12011-023-03684-2

**Published:** 2023-05-05

**Authors:** Shuying Li, Jie Ding, Xiaoxiao Sun, Li Feng, Weihong Zhou, Zhen Gui, Jiangfeng Mao

**Affiliations:** 1 Department of Health Management Center, Nanjing Drum Tower Hospital, Affiliated Hospital of Medical School, Nanjing University, Nanjing, 210000 China; 2 Department of Clinical Laboratory, Children’s Hospital of Nanjing Medical University, Nanjing, 210008 China; 3 Department of Endocrinology, Peking Union Medical College Hospital, Chinese Academy of Medical Sciences, Peking Union Medical College, Beijing, 100730 China

**Keywords:** Insulin resistance, Cardiovascular disease, Triglyceride-glucose index, Selenium, NHANES

## Abstract

Compiling evidence supports that selenium plays a vital role in glucose metabolism. Triglyceride-glucose index (TyG) and triglyceride-glucose-body mass index (TyG-BMI) are commonly used in epidemiologic studies to evaluate insulin resistance and cardiovascular disease (CVD) risks. This study is aimed to investigate the association between whole blood selenium concentration and TyG and TyG-BMI. A total of 6290 participants (age ≥ 20 years) from the National Health and Nutrition Examination Survey (NHANES) 2011–2018 were included. Multiple linear regression models were used to examine the association between blood selenium quartiles and TyG and TyG-BMI. Subgroup analysis stratified by diabetes status was also performed. The adjusted model showed a positive association between TyG and blood selenium concentration (*β* [95%CI] = 0.099 [0.063, 0.134], *p* < 0.001) and TyG-BMI (*β* [95%CI] = 3.185 [2.102, 4.268], *p* < 0.001). The association persisted after stratification by diabetes status (*p* < 0.001). Participants were stratified into four quartiles based on selenium concentration (Q1: 1.08–2.24 μmol/L, Q2: 2.25–2.42 μmol/L, Q3: 2.43–2.62 µmol/L, Q4: 2.63–8.08). Compared with the Q1 group, TyG in the Q3 and Q4 groups was significantly higher (*β* = 0.075 [95%CI 0.039 to 0.112] and *β* = 0.140 [95%CI 0.103 to 0.176], respectively). Additionally, TyG-BMI in the Q2, Q3, and Q4 groups was higher than that in the Q1 group (*β* = 1.189 [95%CI 0.065 to 2.314], *β* = 2.325 [95%CI 1.204 to 3.446], and *β* = 4.322 [95%CI 3.210 to 5.435], respectively). Blood level of selenium was positively associated with TyG and TyG-BMI, indicating that excessive blood selenium may be associated with impaired insulin sensitivity and increased risk of cardiovascular disease.

**Supplementary Information:**

The online version contains supplementary material available at 10.1007/s12011-023-03684-2.

## Introduction

Selenium is an essential trace element for humans, participating in the formation of various selenoproteins, including glutathione peroxidase (GPx), iodothyronine deiodinase (DIO), selenoprotein P (*SELENOP*), selenoprotein S (*SELENOS*), and thioredoxin reductase (Trxr) [1–3]. Early studies suggest that selenium plays a role in insulin mimic and anti-diabetes [4, 5]. Studies have shown that serum selenium levels in diabetic populations are lower than in healthy populations [6]. Furthermore, selenium has been suggested to play a protective role against type 2 diabetes (T2DM) [7, 8], and high levels of selenium may reduce the prevalence of diabetes [9]. In addition, selenium has been shown to prevent atherosclerosis by modulating inflammatory processes, inhibiting oxidative stress, and protecting endothelial cells from apoptosis [10, 11]. However, the relationships between selenium and metabolic diseases are complex, and recent studies have revealed that excessive selenium supplementation may have adverse effects on β-cell function and insulin sensitivity [2]. Higher selenium concentration may interfere with insulin signal transduction, leading to the acceleration of impaired glucose metabolism [12]. Selenium in obesity was negatively associated with body mass index (BMI); however, selenium levels were higher in subjects with metabolic syndrome[13]. Furthermore, a longitudinal study found that a high level of selenium is associated with the development of hypertension [14].

Triglyceride-glucose index (TyG) and triglyceride-glucose-body mass index (TyG-BMI), derived from fasting plasma glucose (FPG) and triglyceride (TG), are two indicators for evaluating insulin resistance(IR) in epidemiological studies [15]. IR contributes to developing CVD in individuals with diabetes and non-diabetes. TyG emerged as a new force, which was shown to be superior to homeostasis model assessment-insulin resistance (HOMA-IR) in evaluating IR, especially for diabetes individuals receiving insulin therapy or without functioning beta cells [35, 36]. TyG and TyG-IBM are positively correlated with insulin resistance (HOMR-IR and HbA1c) [37, 38]. TyG may be helpful to predict the occurrence of DM and it is a useful index reflecting glycemic control for T2DM (AUC = 0.806) [35, 39, 40]. Furthermore, TyG has been proved to be an independent predictor for atherosclerotic cardiovascular diseases (ASCVD) by several studies [41–43]. Since a 10 years follow-up study promulgate a positive correlation between TyG and CVD events (AUC = 0.708), the investigations detecting their relationships emerge in large numbers [43–45]. Elevated TyG is a marker for macro- and microvascular damage [16]. A cross-sectional study from Japan shows a positive correlation between TyG and subclinical atherosclerosis [17].

Since TyG and TyG-BMI are reliable and easy to obtain, they may be effective indicators to demonstrate CVD events in the future for diabetes and non-diabetes individuals. To date, the relationship between blood level of selenium and TyG or TyG-BMI has not been investigated. Therefore, our study aims to investigate the relationship of selenium with TyG and TyG-BMI.

## Methods

### Data Sources

The NHANES (National Health and Nutrition Examination Survey) is a population-based cross-sectional survey designed to collect information on the health and nutrition status of adults and children in the USA. Most of the data in NHANES is freely accessible to researchers worldwide. This investigation pooled data from 2011 to 2018.

### Study Population

**Fig. 1:**
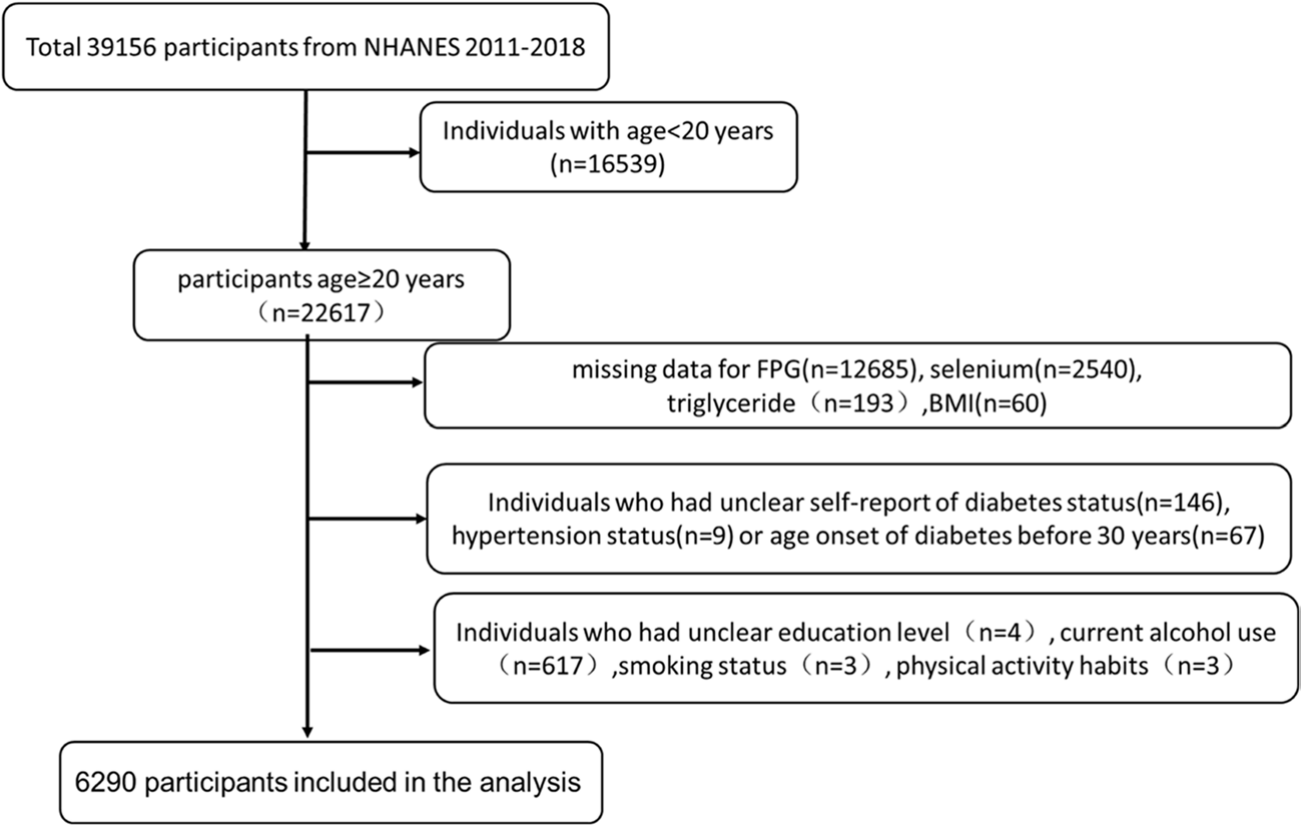
Flow chart for inclusion of participants from NHANES 2011–2018

A total of 39,156 persons were enrolled in NHANES from 2011 to 2018. Participants (*n* = 16,539) with ages younger than 20 years old were excluded. We excluded 12,685, 2540, and 193 individuals due to the absence of data on FPG, whole blood selenium, and TG, respectively. Individuals without self-report diabetes status (*n* = 146) or hypertension status (*n* = 9) were excluded. Besides, 67 persons with diabetes onset age below 30 years were excluded to minimize the confounding factor for type 1 diabetes mellitus. Individuals having no record of education level (*n* = 4), current alcohol use (n = 617), smoking status (*n* = 3), and physical activity habits (*n* = 3) were also excluded. Finally, a total of 6290 participants was included for data analysis (see Fig. 1 for details). The survey protocol was approved by the Institutional Review Board of the Centers for Disease Control and Prevention (CDC). Written consent informs were obtained from all participants.

### Study Variables

The exposure variable in this study is whole-blood selenium. Through inductively coupled plasma mass spectrometry (ICP-MS) and dynamic reaction cell technology (ELAN ® DRC II, Perkin Elmer Norwalk) measures blood selenium concentration using a diluted sample preparation step followed by a whole blood sample. The lower limit of detection (LLOD) is 24.48 μg/L. All data are above LLOD. The index for our investigation was TyG and TyG-BMI. TyG was calculated by the formula of Ln[TG (mg/dL) × FPG (mg/dL)/2] [18], and TyG-BMI was calculated by TyG × BMI [19]. Type 2 diabetes was defined by self-report or FPG ≥ 7.0 mmol/L (126 mg/dL) or glycosylated hemoglobin (HbA1c) ≥ 6.5%. Hypertension was defined by self-report, or the measured systolic blood pressure ≥ 140 mmHg or diastolic blood pressure ≥ 90 mmHg. Smoker was defined as the consumption of more than 100 cigarettes in the whole life [20]. Current alcohol use was defined as the consumption of more than 12 drinks last year [21]. Active physical activity status was defined as a continuously vigorous and intensive activity for at least 10 min in a typical week. Education level was stratified according to whether he or she had attended high school. Sociodemographic variables such as age, sex, and race were extracted from the file named demographic variables. BMI was extracted from the file named body measures. Serum uric acid (SUA) and creatinine (SCr) were extracted from standard biochemistry profiles. Total cholesterol, triglyceride, FPG, HbA1c, and whole blood selenium were extracted from the laboratory data. The selection of covariates in this study was based on a review of relevant literature, and multiple possible covariates related to selenium levels were selected, including age, race, sex, hypertension, smoking, physical activity, alcohol consumption, cholesterol, and kidney function-related indicators (such as serum uric acid and serum creatinine) [22–27].

### Statistical Analyses

All analysis was performed by Empower Stats (Version 4.1) and with the built-in R packages. NHANES sample weights were used as recommended by the National Center for Health Statistics (NCHS). Continuous variables were represented as mean ± SD, and the *p*-value was calculated by weighted linear regression model between T2DM and non-DM groups. Categorical variables were represented as %, and the p-value was calculated by weighted chi-square test between T2DM and non-DM groups. The association between blood level of selenium and TyG or TyG-BMI was evaluated by multiple linear regression analysis models. Participants were stratified to 4 quartiles according to selenium concentration. (Q1:1.08–2.24 μmol/L, Q2: 2.25–2.42 μmol/L, Q3: 2.43–2.62 µmol/L, Q4: 2.63–8.08). Compared with the Q1 quartile group, the association between whole blood selenium and TyG/TyG-BMI in Q2-4 quartile groups by multiple linear regression analysis models. The models were adjusted for age, sex, race, smoker, current alcohol use, education level, physical activity, BMI, HTN, diabetes status, HbA1c, TC, SUA, and Scr. Subgroup analysis, according to diabetic status, was performed. The models for subgroup analysis were not adjusted to diabetes status. Tests for linear trend were performed by entering the median value for each quintile in the models. *P* value < 0.05 is defined as a significant difference.

## Results

The characteristics of the participants were shown in Table 1. Compared with the non-diabetes group, the T2DM group had older age, higher BMI, higher level of SUA and SCr, and lower level of TC. At the same time, they had a higher incidence of hypertension, smoking, current alcohol use, and low education level. The blood level of selenium in the T2DM group was 1.2% higher than that in the non-DM group (*p* = 0.038). TyG and TyG-BMI in the T2DM group were 8.2% and 25.3% higher than those in the non-DM group *p* < 0.001, respectively). Analysis of simple correlation was conducted to evaluated covariates which affect TyG and TyG-BMI. It was found that sex, race, education, physical activity, BMI, HTN, DM, HbA1c, TC, SUA, SCr, and, selenium were associated with TyG and TyG-BMI (Supplementary Table 1).

**Table 1:** Characteristics of participants included in the study

	Total (*n* = 6290)	T2DM (*n* = 1249)	Non-DM (*n* = 5041)	*P*-value
Age(years)	48 ± 17	61 ± 12	46 ± 17	< 0.001
Sex (Male, *n*%)	49.6	54.0	48.9	0.005
Race (*n*%)				< 0.001
Mexican American	8.4	9.9	8.1	
Other Hispanic	6.5	6.6	6.46	
Non-Hispanic White	67.0	61.1	68.0	
Non-Hispanic Black	9.9	13.0	9.4	
Other Race—Including Multi-Racial	8.2	9.5	8.0	
BMI(kg/m^2^)	29.2 ± 7.0	33.6 ± 7.8	28.6 ± 6.6	< 0.001
SUA(μmol/L)	324.8 ± 83.0	344.3 ± 93.3	321.6 ± 80.6	< 0.001
SCr(mg/dL)	0.87 ± 0.34	0.96 ± 0.69	0.86 ± 0.23	< 0.001
TC(mg/dL)	191.8 ± 41.3	180.8 ± 46.5	193.6 ± 40.0	< 0.001
HbA1c(%)	5.6 ± 0.9	7.2 ± 1. 6	5.4 ± 0. 4	< 0.001
FPG(mg/dL)	107.4 ± 30.7	155.8 ± 56.8	99.3 ± 9.8	< 0.001
TG(mg/dL)	120.4 ± 92.4	160.2 ± 125.6	113. 7 ± 83. 6	< 0.001
TyG	8.6 ± 0.7	9.2 ± 0.7	8.5 ± 0.6	< 0.001
TyG-BMI	251. 9 ± 68.6	304. 6 ± 78.7	243.0 ± 62.5	< 0.001
Selenium(μmol/L)	2.48 ± 0.36	2.51 ± 0.38	2.48 ± 0.36	0.038
Smoker (n%)	44.3	50.3	43.3	< 0.001
Active physical activity (n%)	23.7	16.8	24.9	< 0.001
Education at least high school(n%)	86.0	79.1	87.2	< 0.001
Current alcohol use (n%)	55.5	62.4	41.5	< 0.001
HTN(n%)	38.7	70.3	33.4	< 0.001

Continuous variables were represented as mean ± SD, and the *p*-value was calculated by weighted linear regression model between T2DM and non-DM groups. Categorical variables were represented as %, and the *p*-value was calculated by weighted chi-square test between T2DM and non-DM groups. *BMI* body mass index, *SUA* serum uric acid, *SCr* serum creatinine, *TC* total cholesterol, *HbA1c* glycosylated hemoglobin, *FPG* fasting plasma glucose, *TG* triglyceride. TyG: Ln[TG (mg/dL) × FPG (mg/dL)/2]. TyG-BMI: TyG × BMI. Smoker: consumption of more than 100 cigarettes in the whole life. Current alcohol use: consumption of more than 12 drinks during last year. Active physical activity: continuously vigorous and intensive activity for at least 10 min in a typical week. *HTN* hypertension

### Association of Blood Level of Selenium with TyG or with TyG-BMI

There was a positive association of selenium concentration with TyG and TyG-BMI (*β* = 0.219 and 8.047, 95%CI = 0.174 to 0.265 and 3.350 to 12.744). After fully adjusted, every 1 μmol/L increment in selenium concentration corresponds to 0.099 and 3.185 increase in TyG and TyG-BMI, respectively. In the T2DM group, after being adjusted for multiple covariates, every 1 μmol/L increment in selenium concentration corresponds to 0.137 and 5.332 increases in TyG and TyG-BMI, respectively. In the non-DM group, the corresponding increase in TyG and TyG-BMI was 0.090 and 2.802, respectively. (Details in Table 2).

**Table 2:** Association between TyG/TyG-BMI and selenium

	*β*(95% CI) *p*-value		
	Model 1	Model 2	Model 3
TyG			
Total	0.219 (0.174, 0.265) < 0.001	0.188 (0.144, 0.233) < 0.001	0.099 (0.063, 0.134) < 0.001
T2DM	0.171 (0.063, 0.279) 0.002	0.146 (0.038, 0.253) 0.008	0.137 (0.049, 0.225) 0.002
Non-DM	0.206 (0.161, 0.252) < 0.001	0.178 (0.133, 0.223) < 0.001	0.090 (0.051, 0.129) < 0.001
TyG-BMI			
Total	8.047 (3.350, 12.744) < 0.001	7.801 (3.105, 12.498) 0.001	3.185 (2.102, 4.268) < 0.001
T2DM	4.003 (− 7.508, 15.514) 0.496	2.800 (− 8.049, 13.649) 0.613	5.332 (2.355, 8.308) < 0.001
Non-DM	6.951 (2.122, 11.780) 0.005	6.624 (1.783, 11.465) 0.007	2.802 (1.657, 3.947) < 0.001

Model 1: Unadjusted. Model 2: Adjusted for age and sex. Model 3: Adjusted for age, sex, race, smoker, current alcohol use, education, physical activity, BMI, HTN, DM, HbA1c, TC, SUA, and SCr. DM was not adjusted in the subgroup analysis. BMI was not adjusted in the evaluation of the correlation between TyG-BMI and selenium

### Association Between TyG, TyG-BMI, and Blood Selenium Concentration Quartiles

**Fig. 2:**
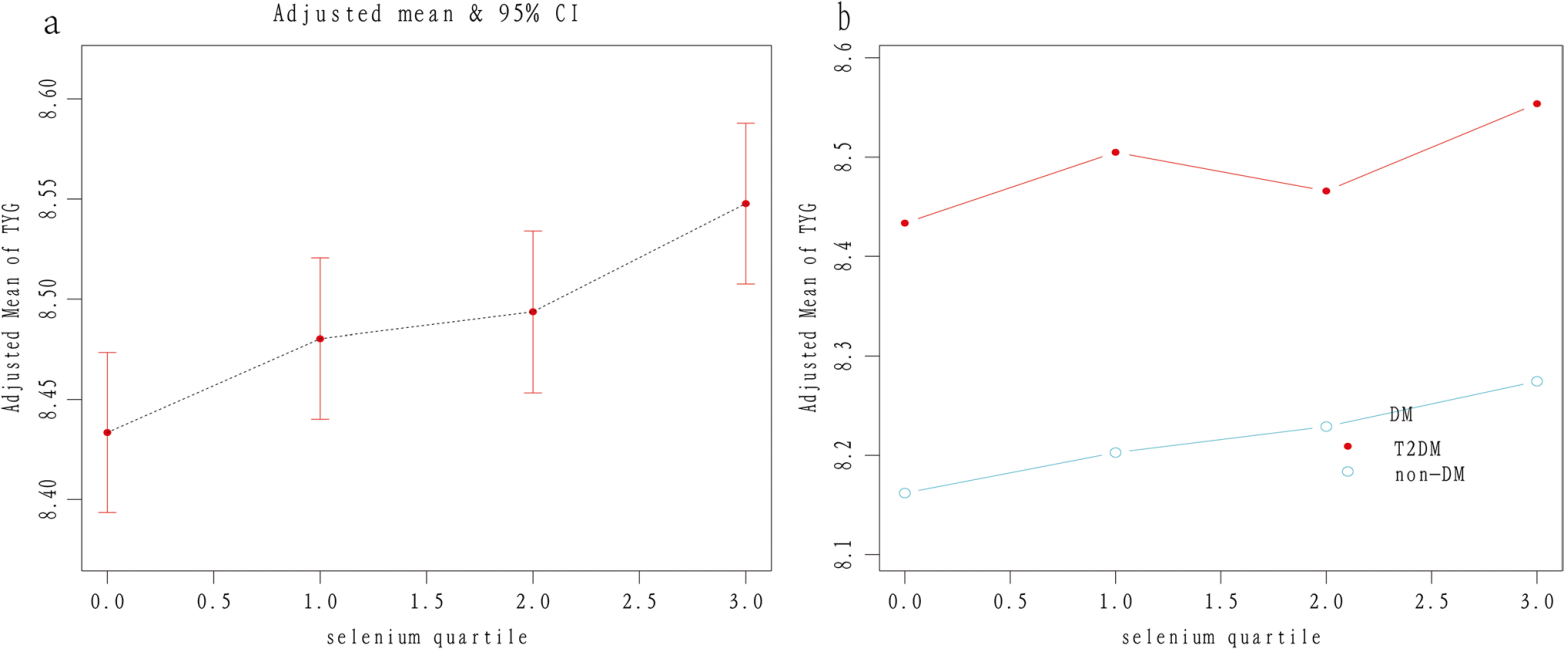
Increasing TyG in four quartiles with higher selenium concentration. Note a: TyG increased with the selenium quartile increasing (*P*-trend < 0.001). Note b: trend for T2DM and non-DM respectively (*P*-trend* < 0.001, *P*-trend# < 0.01)

Participants were stratified into 4 quartiles according to blood selenium concentration (Q1:1.08–2.24 μmol/L, Q2: 2.25–2.42 μmol/L, Q3: 2.43–2.62 µmol/L, Q4: 2.63–8.08). Compared with the Q1 quartile group, TyG in Q3 and Q4 quartiles was significantly higher with the *β*’s of 0.075 and 0.140 (both *p* < 0.001). In the T2DM group, TyG in Q4 was higher than in Q1 with *β* = 0.187 (*p* < 0.001). In the non-DM group, TyG in Q3 and Q4 was higher than in Q1 with *β* = 0.082 and 0.130 (both *p* < 0.001). The *p*-trend for 4 quartiles classified by diabetes status was statistically significant (*p* < 0.001) (details in Table 3 and Fig. 2).

**Table 3:** Association between TyG and categorical selenium

Diabetes status	*β*(95% CI) *p*-value		
	Model 1	Model 2	Model 3
Selenium(μmol/L) group			
Q1(1.08–2.24)	Ref	Ref	Ref
T2DM	Ref	Ref	Ref
Non-DM	Ref	Ref	Ref
Q2(2.25–2.42)	0.086 (0.038, 0.134) < 0.001	0.079 (0.033, 0.126) < 0.001	0.036 (− 0.001, 0.072) 0.058
T2DM	0.113 (− 0.008, 0.235) 0.068	0.106 (− 0.014, 0.226) 0.083	0.092 (− 0.006, 0.191) 0.066
Non-DM	0.091 (0.044, 0.139) < 0.001	0.084 (0.038, 0.130) < 0.001	0.031 (− 0.009, 0.071) 0.126
Q3(2.43–2.62)	0.158 (0.110, 0.206) < 0.001	0.139 (0.092, 0.185) < 0.001	0.075 (0.039, 0.112) < 0.001
T2DM	0.050 (− 0.071, 0.171) 0.416	0.034 (− 0.086, 0.154) 0.578	0.027 (− 0.071, 0.125) 0.586
Non-DM	0.185 (0.138, 0.232) < 0.001	0.163 (0.117, 0.209) < 0.001	0.082 (0.043, 0.122) < 0.001
Q4(2.63–8.08)	0.282 (0.235, 0.329) < 0.001	0.263 (0.218, 0.308) < 0.001	0.140 (0.103, 0.176) < 0.001
T2DM	0.266 (0.153, 0.379) < 0.001	0.239 (0.127, 0.351) < 0.001	0.187 (0.095, 0.279) < 0.001
Non-DM	0.266 (0.219, 0.313) < 0.001	0.247 (0.202, 0.293) < 0.001	0.130 (0.091, 0.170) < 0.001
* P*-trend	< 0.001	< 0.001	< 0.001
* P*-trend*/*P*-trend#	< 0.001/ < 0.001	< 0.001/ < 0.001	< 0.001/ < 0.001

Model 1: Unadjusted. Model 2: Adjusted for age and sex. Model 3: Adjusted for age, sex, race, smoker, current alcohol use, education, physical activity, BMI, HTN, DM, HbA1c, TC, SUA, and SCr. DM was not adjusted in the subgroup analysis. *P*-trend was calculated among the categorical selenium groups. *P*-trend # stands for the non-DM group. *P*-trend * stands for the T2DM group

**Fig. 3:**
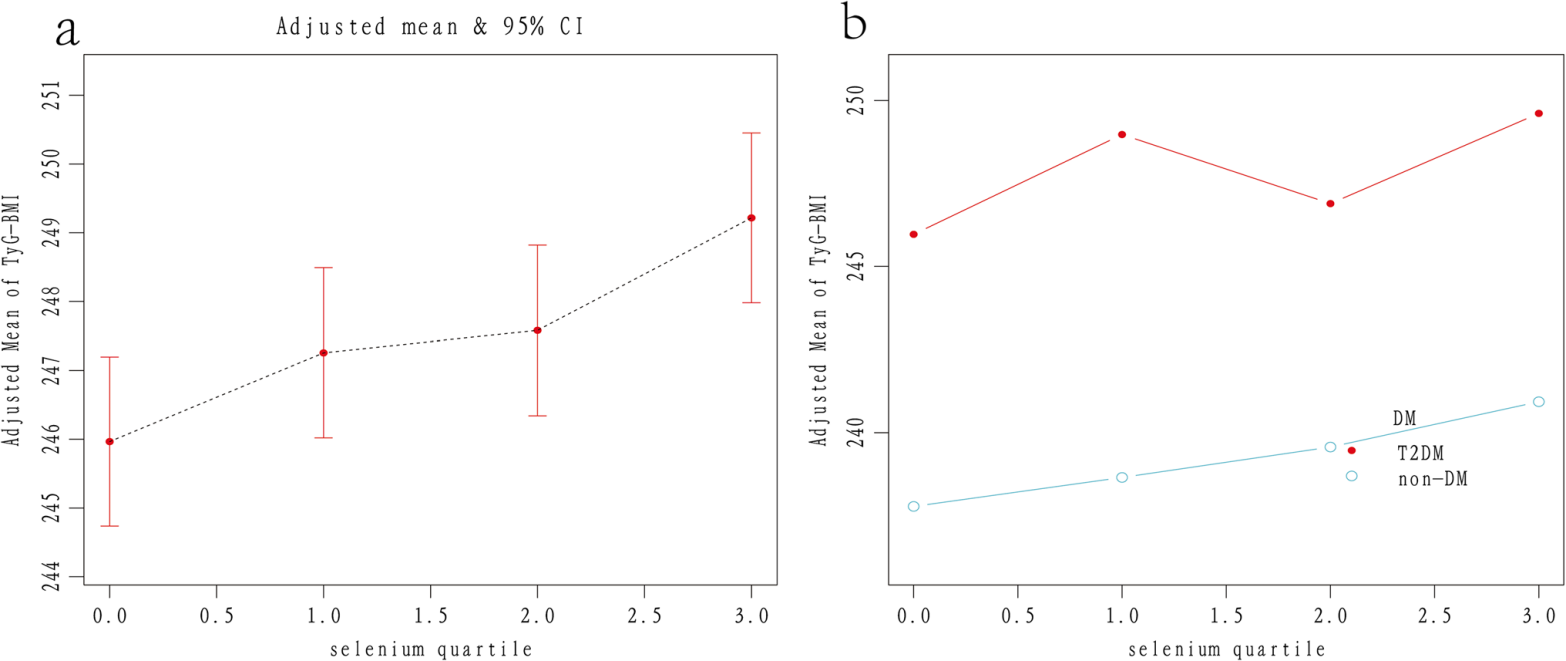
Increasing TyG-BMI in four quartiles with higher selenium concentration. Note a: TyG-BMI increased with the selenium quartile increasing *(P*-trend < 0.001). Note b: trend for T2DM and non-DM respectively (*P*-trend* < 0.001, *P*-trend# < 0.01)

Compared with the Q1 quartile group, TyG-BMI in Q2, Q3, and Q4 were significantly higher with *β* = 1.189, 2.325, and 4.322 (both *p* < 0.001), respectively. In the T2DM group, the TyG-BMI in Q2 and Q4 was higher than in Q1 with *β* = 4.172 and 6.820 (*p* = 0.014 and *p* < 0.001). In the non-DM group, TyG-BMI in Q3 and Q4 was higher than in Q1 with *β* = 2.533 and 3.899 (both *p* < 0.001). The *p*-trend for 4 quartiles classified by diabetes status was statistically significant (*p* < 0.001) (Details in Table 4 and Fig. 3).

**Table 4:** Association between TyG-BMI and categorical selenium

Diabetes status	*β*(95%CI) *p*-value		
	Model 1	Model 2	Model 3
Selenium(μmol/L) group			
Q1(1.08–2.24)	Ref	Ref	Ref
T2DM	Ref	Ref	Ref
Non-DM	Ref	Ref	Ref
Q2(2.25–2.42)	4.453 (− 0.498, 9.404) 0.078	4.414 (− 0.515, 9.344) 0.079	1.189 (0.065, 2.314) 0.038
T2DM	4.921 (− 8.063, 17.906) 0.458	4.794 (− 7.327, 16.915) 0.438	4.172 (0.849, 7.494) 0.0140
Non-DM	5.225 (0.201, 10.249) 0.042	5.110 (0.091, 10.129) 0.046	0.848 (− 0.326, 2.023) 0.157
Q3(2.43–2.62)	7.876 (2.971, 12.780) 0.002	7.999 (3.103, 12.895) 0.001	2.325 (1.204, 3.446) < 0.001
T2DM	− 7.337 (− 20.231, 5.557) 0.265	− 7.380 (− 19.471, 4.712) 0.232	2.042 (− 1.264, 5.349) 0.226
Non-DM	11.136 (6.161, 16.111) < 0.001	10.982 (6.000, 15.964) < 0.001	2.533 (1.364, 3.703) < 0.001
Q4(2.63–8.08)	11.744 (6.917, 16.570) < 0.001	11.865 (7.047, 16.683) < 0.001	4.322 (3.210, 5.435) < 0.001
T2DM	11.133 (− 0.917, 23.184) 0.070	9.002 (− 2.332, 20.335) 0.120	6.820 (3.708, 9.932) < 0.001
Non-DM	10.190 (5.249, 15.130) < 0.001	10.118 (5.172, 15.063) < 0.001	3.899 (2.727, 5.071) < 0.001
*P*-trend	< 0.001	< 0.001	< 0.001
*P*-trend*/*P*-trend#	0.119/ < 0.001	0.201/ < 0.001	< 0.001/ < 0.001

Model 1: Unadjusted. Model 2: Adjusted for age and sex. Model 3: Adjusted for age, sex, race, smoker, current alcohol use, education, physical activity, HTN, DM, HbA1c, TC, SUA, and SCr. DM was not adjusted in the subgroup analysis. *P*-trend was calculated among the categorical selenium groups. *P*-trend * was for the T2DM group. P-trend# was for the non-DM group

## Discussion

TyG and TyG-BMI are two novel indicators to evaluate insulin resistance and cardiovascular disease risks in epidemiological studies. Our investigation shows that blood selenium concentration is positively associated with TyG and TyG-BMI. This association still exists despite T2DM or not. This is the first study to reveal a close relationship between selenium concentration and TyG or TyG-BMI, what’s more provides epidemiological evidence that the sensitivity of selenium rich people to insulin may be impaired.

The researchers combined the data from multiple studies to conclude that a blood selenium concentration of 1.0–1.2 μmol/l is sufficient to maximize GPx and SELENOP, as well as possibly other selenoproteins [28]. In our study, the average concentration of selenium in the American population (2.4 μmol/L) is relatively higher than in other countries and regions (0.82–1.41 μmol/L) [29–34]. Our investigation demonstrated that excessive blood selenium is associated with more severe insulin resistance indicated by TyG and TyG-BMI. After being adjusted by all compounding factors, the effect of selenium levels on TyG and TyG-BMI was very mild (For every 1 μmol/L increment in selenium concentration, TyG and TyG-BMI would increase by 0.15 SD (0.099/0.671) and 0.05 SD (3.185/68.572), respectively. Our results indicate that supplementation of selenium should not be recommended for people replete with selenium.

In this study, TyG and TyG-BMI were used as surrogates to explore the relationship between insulin sensitivity and blood level of selenium. Previous studies showed a beneficial effect of selenium supplementation on glucose metabolism in populations with low selenium [5, 35]. Both animal and epidemiologic investigations have found that selenium deficiency is associated with decreased insulin sensitivity, which could be improved by appropriate supplementation [36–40]. However, recent emerging evidence showed that excessive selenium may have a detrimental effect on insulin sensitivity. Cardoso et al. (2021) found that the serum concentration of selenium in the selenium-replete population in the United States was positively correlated with fast insulin level and insulin resistance (HOMA-IR) [41]. A meta-analysis, collecting data from five experimental studies, found that selenium supplementation (200 μg/day) increased the risk of diabetes by 11% (RR 1.11, 95% CI 1.01–1.22) [42]. Although the comprehensive influences of selenium on glucose metabolism and insulin sensitivity are not conclusive, our investigation, by using indexes of TyG and TyG-BMI, indicated a detrimental effect on glucose metabolism by a higher level of selenium. We noticed that in the T2DM population, higher selenium concentration is associated with poorer glycemic control (high TyG and TyG-BMI). In a word, all these data revealed that deficient or excessive selenium would bring adverse effects on glycemic metabolism.

Considering the relationship between TyG and TyG-BMI and CVD, we speculate that selenium may be related to CVD, although we did not use CVD as the outcome of our research. The relationship and underlying mechanism between selenium concentration and CVD risk had not been fully investigated. Selenium supplementation showed some anti-atherosclerosis effects in cell lines and animal models researches [10]. It is estimated that increasing selenium concentration is associated with reducing CVD risks when the selenium concentration was 0.70–1.84 μmol/L [43–45]. Above this range, the anti-atherosclerosis effect of selenium could not be observed [46]. Consistent with the above evidence, our study revealed an association between higher blood selenium concentration and TyG and TyG-BMI in the population with excessive blood selenium. It is conjectured a U-shaped curve relationship between selenium concentration and CVD risks.

The biological form of selenium consists essentially of the amino acid selenocysteine which is incorporated into selenoproteins, such as SELENOP, GPx, and Trxr.. These molecules exert pivotal anti-oxidation effects. These selenoproteins are linked to the insulin signaling pathway and insulin secretion pathway as an antioxidant [47]. The activity of some key enzymes in insulin signaling was inhibited by SELENOP, leading to reduced insulin sensitivity [48]. Overexpression of GPx1 would impair the insulin signaling pathway by inhibiting ROS or hydrogen peroxide (H2O2) production, weakening the activation of protein tyrosine phosphatase 1B, and inhibiting the phosphorylation of insulin receptor and protein kinase B [49, 50]. Abnormal GPx3 expression may lead to the accumulation of local reactive oxygen species in adipose tissue and increase the risk of diabetes [51].

This study has some limitations. First, due to limited data from the US population, we could not figure out the whole picture reflecting the association between selenium concentration and insulin sensitivity since the population with selenium deficiency was not included. Second, a cross-sectional designed study could not conclude a causal relationship between selenium concentration and TyG and TyG-BMI. Thirdly, the study is in the lack of data on selenium intake in the population under investigation. High-quality, interventional and prospective studies are required to clarify the effects of selenium on diabetes development.

In conclusion, selenium concentration is positively correlated with TyG and TyG-BMI in the population with replete selenium, indicating detrimental effects of excessive selenium intake on glucose metabolism and CVD risks. The underlying mechanism needs to be further investigated.

### Supplementary Information

Below is the link to the electronic supplementary material.

Supplementary file 1 (DOCX 19 KB)

## Data Availability

The original contributions presented in the study are included in the article material, further inquiries can be directed to the corresponding authors.
