# Supplementary material for: Selenium Concentration Is Positively Associated with Triglyceride-Glucose Index and Triglyceride Glucose-Body Mass Index in Adults: Data from NHANES 2011–2018
Source: Biol Trace Elem Res. 2023 May 5;202(2):401–9. doi: 10.1007/s12011-023-03684-2 (PMC10764531; doi:10.1007/s12011-023-03684-2)
Supplement: Supplementary file 1 — (DOCX 19 KB) [file 12011_2023_3684_MOESM1_ESM.docx]

Supplementary Table 1: Analysis of simple correlation evaluating covariates which affect TyG and TyG-BMI.

| Items | TyG (β[95% CI]p-value) | TyG-BMI (β[95% CI]p-value) |
| --- | --- | --- |
| Age（years） | 0.008 (0.007, 0.009) <0.001 | 0.391 (0.291, 0.491) <0.001 |
| Sex (Male, n%) | -0.194 (-0.226, -0.161) <0.001 | 0.531 (-2.859, 3.921) 0.759 |
| Race (n%) |  |  |
| Other Hispanic | -0.115 (-0.201, -0.029) 0.008 | -15.604 (-24.428, -6.781) <0.001 |
| Non-Hispanic White | -0.118 (-0.178, -0.057) <0.001 | -15.341 (-21.520, -9.162) <0.001 |
| Non-Hispanic Black | -0.407 (-0.484, -0.330) <0.001 | -7.876 (-15.784, 0.032) 0.051 |
| Other Race - Including Multi-Racial | -0.093 (-0.174, -0.013) 0.023 | -31.899 (-40.178, -23.621) <0.001 |
| BMI（kg/m^2^） | 0.029 (0.027, 0.031) <0.001 | 9.452 (9.383, 9.522) <0.001 |
| SUA(μmol/L) | 0.002 (0.002, 0.002) <0.001 | 0.261 (0.241, 0.280) <0.001 |
| SCr（mg/dL） | 0.128 (0.079, 0.177) <0.001 | 1.654 (-3.362, 6.669) 0.518 |
| TC（mg/dL） | 0.189 (0.174, 0.204) <0.001 | 4.386 (2.797, 5.975) <0.001 |
| HbA1c（%） | 0.326 (0.310, 0.342) <0.001 | 25.719 (23.995, 27.444) <0.001 |
| T2DM (%) | -0.730 (-0.773, -0.686) <0.001 | -61.567 (-66.150, -56.985) <0.001 |
| Selenium(μmol/L) | 0.219 (0.174, 0.265) <0.001 | 8.047 (3.350, 12.744) <0.001 |
| Smoker (n%) | -0.142 (-0.175, -0.108) <0.001 | -4.369 (-7.779, -0.959) 0.012 |
| Active physical activity (n%) | -0.088 (-0.121, -0.055) <0.001 | -20.133 (-23.508, -16.759) <0.001 |
| Education at least high school(n%) | -0.170 (-0.218, -0.122) <0.001 | -6.268 (-11.152, -1.385) 0.012 |
| Current alcohol use (n%) | -0.088 (-0.121, -0.055) <0.001 | -20.133 (-23.508, -16.759) <0.001 |
| HTN(n%) | -0.328 (-0.361, -0.294) <0.001 | -35.807 (-39.172, -32.441) <0.001 |
